# Construction of a specialized integrated simulation platform for molecule screening based on scientific computing workflow engine

**DOI:** 10.1038/s41598-023-42913-5

**Published:** 2023-09-20

**Authors:** Chengqiu Gou, Jifeng Li, Yufeng Li, Jian Liu, Shicao Zhao, Yonghao Xiao, Bowen Duan

**Affiliations:** 1 Institute of Computer Application, China Academy of Engineering Physics, Sichuan, 621000 China; 2 Institute of Chemical Materials, China Academy of Engineering Physics, Sichuan, 621000 China

**Keywords:** Chemistry, Engineering

## Abstract

Numerical simulation is an efficient tool for evaluation and prediction of material properties and behavior in many industrial domains such as the development of novel materials and medicines. For numerical studies of complex processes or systems with high fidelity, various data processing tools, modeling and simulation programs are typically involved, desiring an integrated platform that can effectively manage the collaboration of such software resources and the execution of the underlying simulation workflow for efficiency purpose. Such a platform could be practically built with a scientific computing workflow engine that focuses on the automatic scheduling and execution of a batch of interrelated computing tasks. In this work, the main procedures on construction of a specialized integrated simulation platform for material research based on a general purpose scientific computing workflow engine named HSWAP is introduced in detail, and its application to molecule screening process of energetic materials is demonstrated. Due to the flexibility and the extensibility of the platform, the work could be handily extended to the screening of other materials such as protein to find optimized protein structures or high entropy alloys to find the best configuration of component contents, as well as other application scenarios such as geometry optimizations of complex structures.

## Introduction

In practice, an engineering simulation job often requires the extensive collaboration of a series of data processing tools, modeling and simulation software programs with close dependence of data and control flow. With the help of scientific workflow techniques, data and software resources involved in the modeling, simulation and analysis processes of a simulation job could be effectively managed and the tasks comprising the job could be automatically executed^1–4^. Scientific workflow techniques have become a research hotspot in computer application and other fields^5–11^. Scientific workflow techniques provide users with a convenient access to the underlying computing environment by hiding the details such as the job submission and data transmission, resulting in dramatic improvement of the computing efficiency. Scientific workflow involves key technologies such as workflow model^12^, workflow representation^13^, workflow description language^14,15^, task scheduling^16–22^, fault tolerance^23–28^ and data provenance^25,29–37^. To benefit from the workflow techniques for meeting the huge demand of complex simulation tasks in academia and industry, a couple of mature scientific workflow management systems have been developed, such as Kepler^26^, PEAGUS^27^, TRIANA^38^, TAVERNA^39^, GALAXY^40^, FlowEngine^41^ and ASKALON^28^, etc. These systems focus on their respective application fields. For example, Kepler is mainly used in process management in biology, astronomy and social ecology; Pegasus is mainly applied to research fields such as bio-informatics and aerospace. As these scientific workflow management systems are targeted at certain specialized application fields, they are only suitable for process management of scientific computing and scientific experiments in respective fields, lacking the flexibility and scalability for developing integrated platforms that satisfy specific user needs.

To facilitate the combined use of various numerical simulation tools and programs deployed in a distributed heterogeneous super-computing environment for conducting an integrated “design-modeling-calculation-analysis-optimization” cycle of simulation task, a scientific computing workflow engine HSWAP (HPC Scientific Workflow Application Platform)^42,43^ has been developed by Chinese Academy of Engineering Physics using scientific workflow techniques. With the capability of flexible integrating of interconnected software tools and programs, automatic scheduling and executing of interrelated simulation tasks, HSWAP offers users a highly efficient way of constructing an integrated domain-specific platform to perform numerical simulations for complex engineering problems.

Molecule screening process refers to the numerical process that finds the most satisfactory molecule structures with desiring performances from a large set of feasible molecules built with given molecular skeletons and functional groups. It is characterized by large computing scale, numerous calculation steps, and various calculation software, and is typically handled with high throughput screening (HTS) techniques, which are approaches that able to perform tens of tests per day^44–47^. With HTS techniques a researcher can quickly conduct a huge amount of numerical tasks, with each task has similar resource requirements for computing and similar data input and output, and rapidly obtain a large amount of information. By performing massive computation processes in parallel, HTS can rapidly discover materials with desired components, structures and functions. To perform an effective molecule screening process with HTS techniques, a specialized integrated simulation platform is required.

The paper is structured as follows: in Section “Construction of domain-specific platform based on HSWAP” the main procedures for constructing a domain-specific integrated simulation platform based on HSWAP are introduced, followed by an application to build a molecule screening platform for energetic materials in Section “Application to construction of molecule screening platform for design of energetic materials”, and finally in Section “Conclusions” some conclusions are made.

## Construction of domain-specific platform based on HSWAP

HSWAP is a scientific computing workflow engine for easily building an integrated simulation platform that enables users solving complex engineering problems efficiently in high performance computing (HPC) environment without the knowledge of involved hardware and software computing resources. In HSWAP a complete simulation work is referred to as a job consisting of several interrelated tasks, with each task being encapsulated as a component, which usually represents an executable software program. A component has certain user-defined attributes such as the hardware resources used, the operating parameters, the input and output settings. Between components there may exist data dependency, which means that the output of one component may be used as the input of the other one. The relationship of the interrelated tasks and their execution sequence is generally called a workflow and described by a directed acyclic graph (DAG).

**Figure 1:**
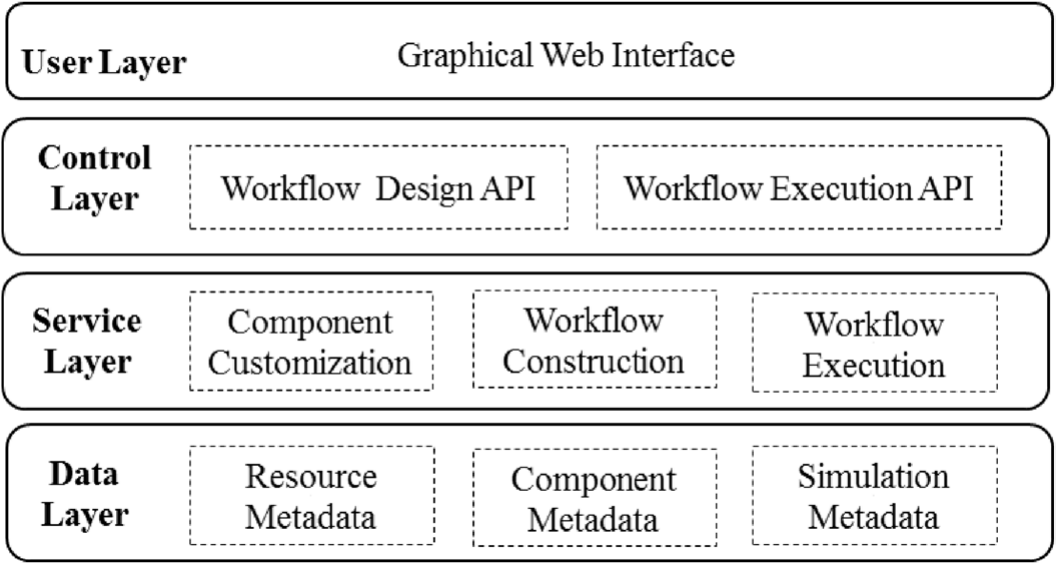
Architecture of HSWAP.

The overall architecture of HSWAP is shown in Fig. 1. The data layer and service layer are the core of the platform. The data layer is responsible for managing various metadata of the platform. The service layer provides functions such as component management, workflow creation, and workflow execution. The control layer provides workflow design API and workflow execution API, providing backend support for users to visually edit and control workflows on the browser side.

The key procedures to construct a domain-specific simulation platform with HSWAP are (1) Construction of the workflow in the backend based on the operational sub-processes involved in the simulation work. (2) Implementation of a customized graphical user interface (GUI) in the frontend based on user requirements. (3) Connect the frontend GUI to the backend workflow. The main steps are listed as below.

1. Identification of the domain-specific process. For a specific domain problem, find out the solution procedures and the detailed operational sub-processes involved. Formulate a diagram of the workflow with each sub-process being represented as a node and the mutual relationship described by a directed edge. Each node has a single responsibility and is executed by using one software program. Interlinked nodes communicate with data or files.
2. Construction of the customized components. Each node in the workflow is encapsulated as a component in HSWAP, which is constructed based on the implementation details of the node.
3. Construction of the customized workflow. The workflow is constructed by instantiation of the components by dragging built-in component templates from the component library based on the dependency of interlinked sub-processes.
4. Implementation of a customized GUI. A customized GUI with good user experience could be implemented easily from a customization tool for GUI provided in HSWAP.

As the GUI is closely dependent on the specific application scenario, here we focus on the introduction of the first three steps listed above.

### Identification of domain-specific process

**Figure 2:**
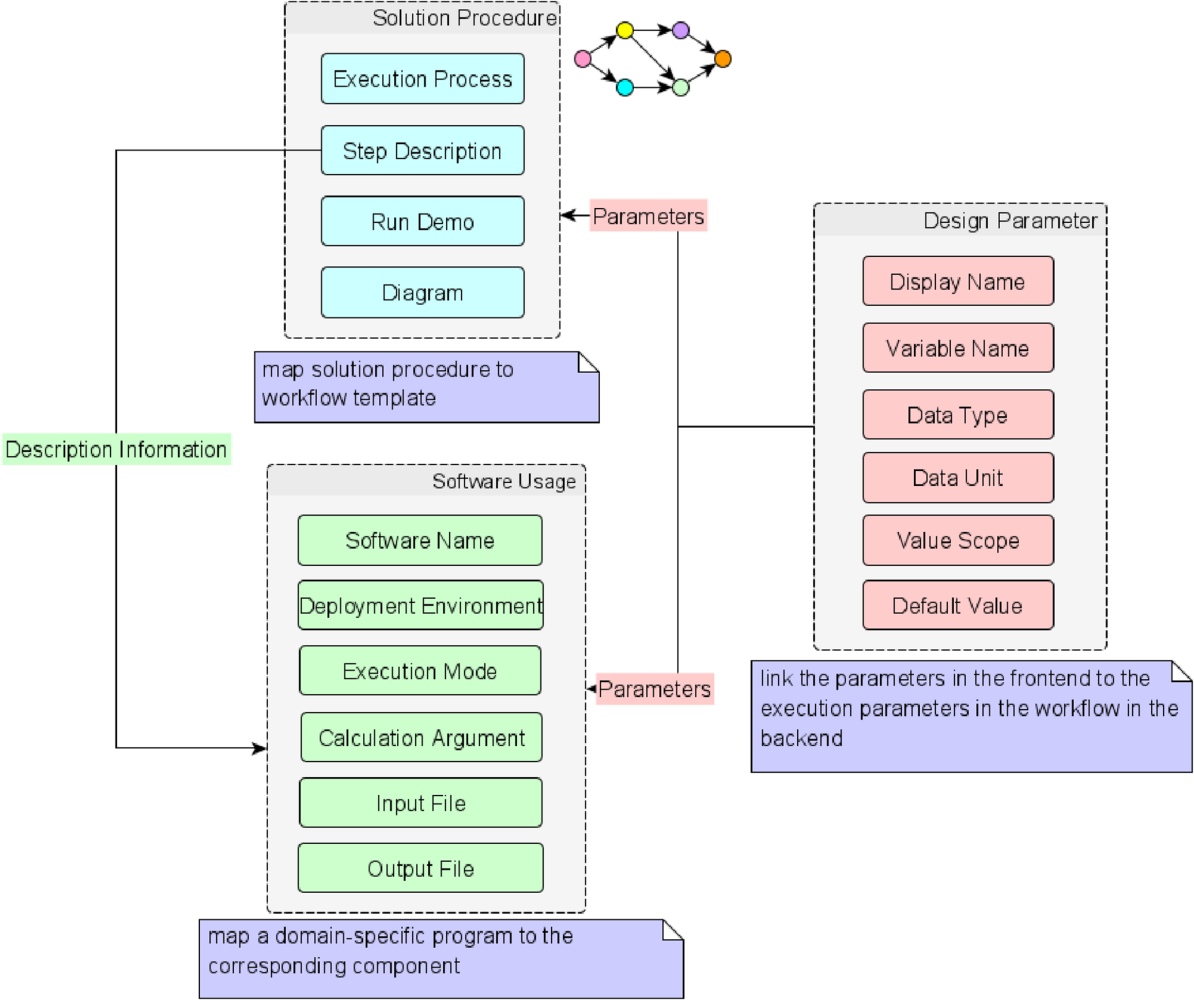
Standard template for user requirement collection.

The main purpose in this step is to collect the actual requirements of domain users and provide an input for the customization of components and workflow. To improve the communication efficiency, a standard template for collecting user’s requirements is designed. The template consists of three parts, namely the software usage part, the solution procedure part and the design parameters part as shown in Fig. 2. The software usage part collects the execution information of the software programs, such as the name of the program, the command and arguments to call the program as well as the input and output of the program. It is mainly used to map a domain-specific program to the cor-responding component that calls it. The solution procedure part collects the detailed solution steps for the engineering problem, such as the identification of all the operational sub-processes in the workflow and the execution details for each sub-process. This part is used to map the solution procedure to the workflow template. The design parameters part collects the design parameters in the UI, such as the name, unit of the parameters and the value range. This part is used to link the parameters in the frontend to the execution parameters in the workflow in the backend.

### Interactive customization of components

Component model have hierarchical structures and are error-prone for manually implementation. For improvement of the efficiency of component customization, an interactive tool for component customization is developed according to the dependency of hierarchical structures of component model.

**Figure 3:**
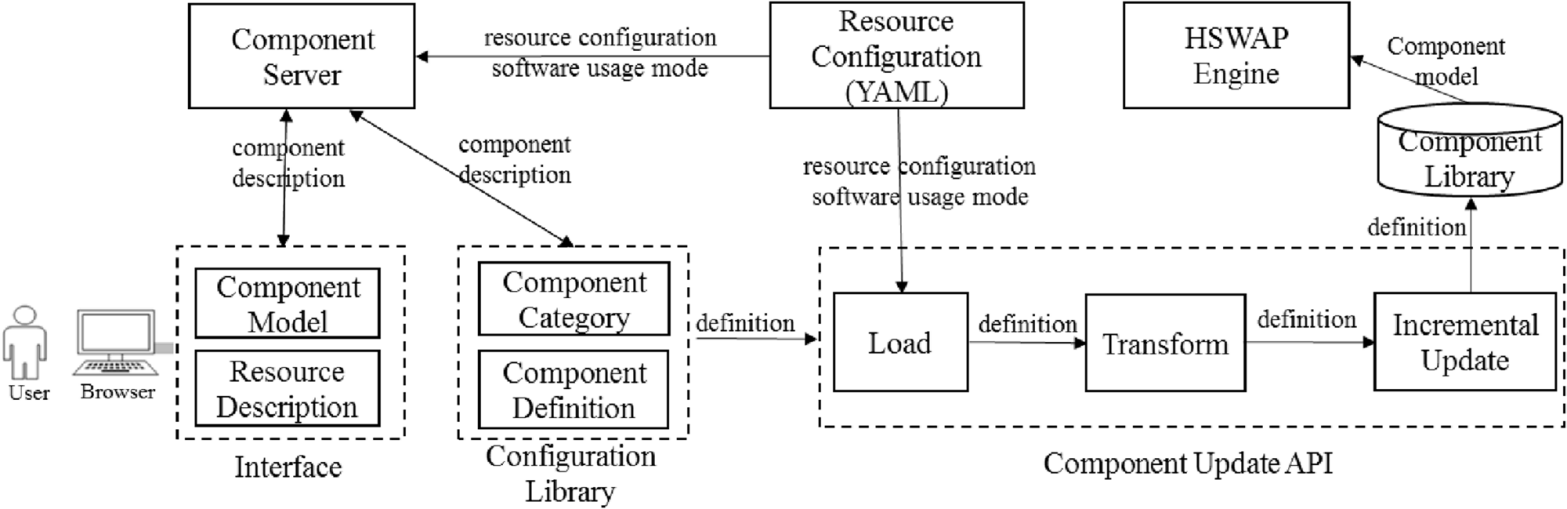
Interactive customization of components.

The main procedures for component customization are illustrated in Fig. 3. The component customization server reads in the configuration of computing resources and displays the information of the execution mode and runtime dependency for users to choose. Users complete the configuration data of the component in the component customization tool, and save the description of the component to the component library with the component customization server, and make an incremental update of the component in the library through the loading, parsing and transformation processes. The component is automatic generated, saved and validated, effectively avoiding the inconsistency issues of component definition.

**Figure 4:**
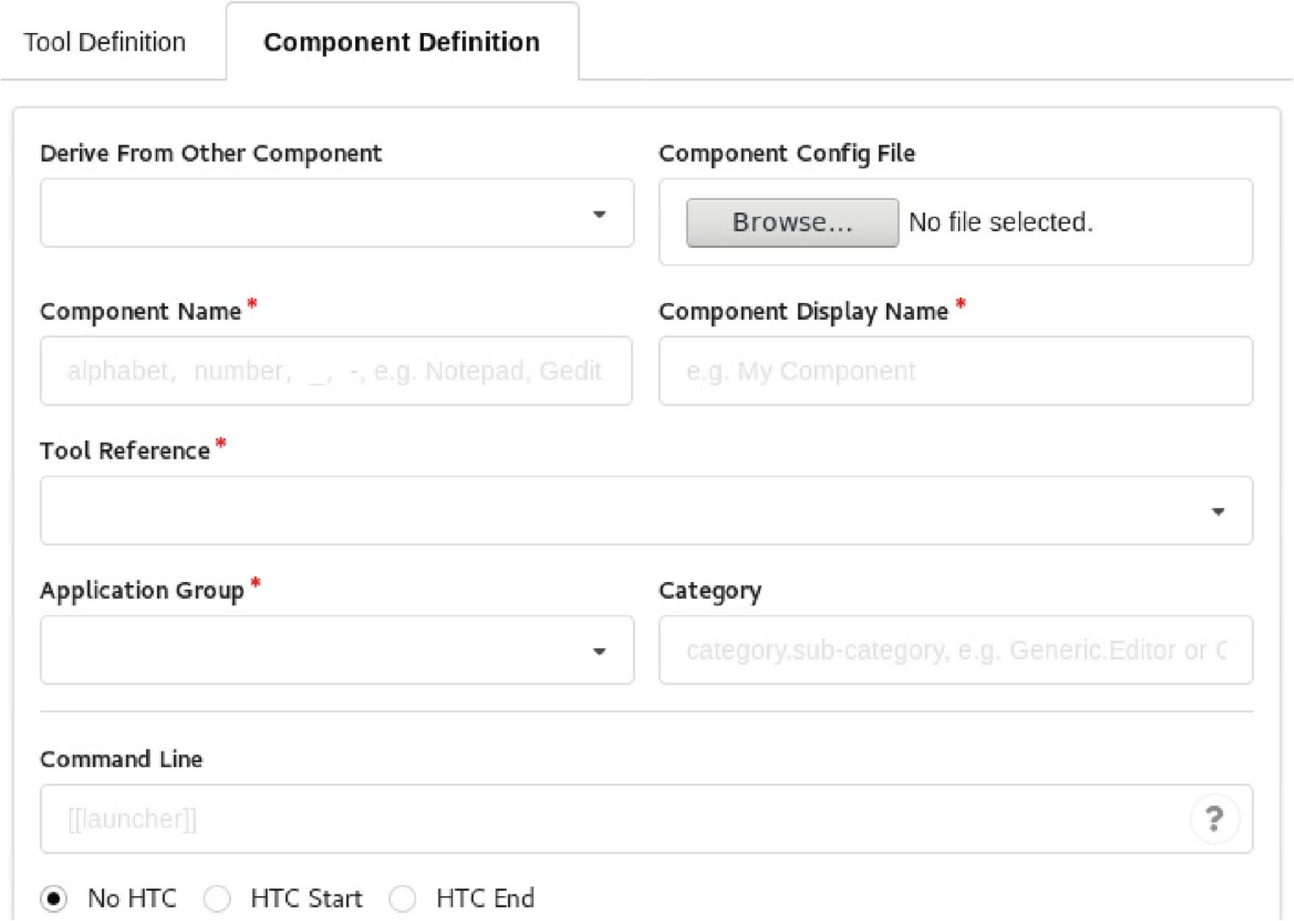
GUI of the auxiliary tool for component customization.

With this auxiliary tool, developers could efficiently construct a customized component by simply filling in an online form for the attributes of underlying computing program, as depicted in Fig. 4, which could avoid the error-prone manual procedure for component configuration, and conceal the complex implementation details for software integration, leading to efficiency improvement for component construction process.

**Figure 5:**
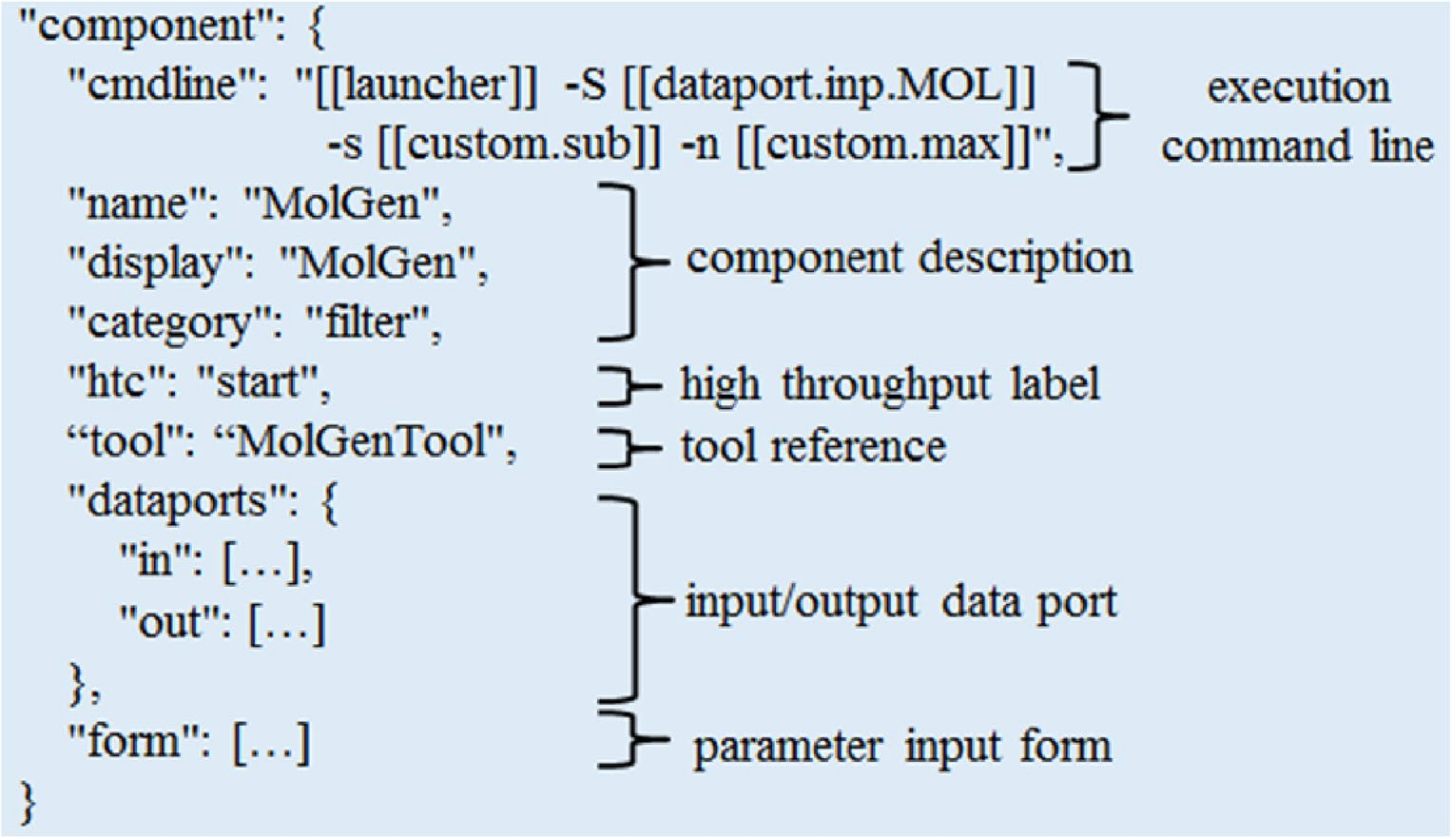
Component description.

The customized component generated through this auxiliary tool is saved in JSON format, as is illustrated in Fig. 5. The component MolGen is descried with several attributes. The cmdline attribute describes the command line for execution of the software or tools associated with this component. The name and display attributes are used to specify the names of the component stored in the backend and displayed in the frontend, respectively. The category attribute represents the grouping information of the component. The htc attribute indicates whether the component is executed in a high-throughput computation. The tool attribute is a reference of the underlying computational software or tool associated with this component. The dataports attribute defines the input and output data ports of the component. The form attribute refers to a form for collecting user input parameters.

### Interactive customization of workflow

HSWAP provides functionalities for workflow viewing and editing. In the GUI for workflow editing, nodes in the workflow could be interactively added by dragging components from the component library to the workflow canvas. Connection between a pair of nodes is represented by a directed edge. Attributes for each node and edge are configurable. When operations that could change the topology of the DAG of the workflow such as deleting nodes or edges are performed in the workflow canvas, the corresponding information is sent to the backend server synchronously for data updating to ensure the data consistency between the front and the back ends.

## Application to construction of molecule screening platform for design of energetic materials

In this section the procedures for construction of domain-specific platform introduced in Section “Construction of domain-specific platform based on HSWAP” is applied to build a molecule screening platform for energetic materials. Traditional methods for development of novel energetic materials mainly rely on intensive experiments, which are suffered from long design cycle, low working efficiency, high material consumption and high safety risk, thus innovative techniques are demanded for resolving these issues. In this work, we attempt to accelerate the development process of energetic materials with numerical methods through an integrated platform constructed by HSWAP. The main steps are as follows: firstly, identify the detailed business process for the molecule screening of energetic materials; then, build components based on the component model of the HSWAP. Each component represents a software program or tool involved in the processes and constructs the workflow with the components; finally, develop a customized GUI for energetic material designers to conduct rapid building and screening of desired molecules for energetic materials.

### Identification of molecule screening process

**Figure 6:**
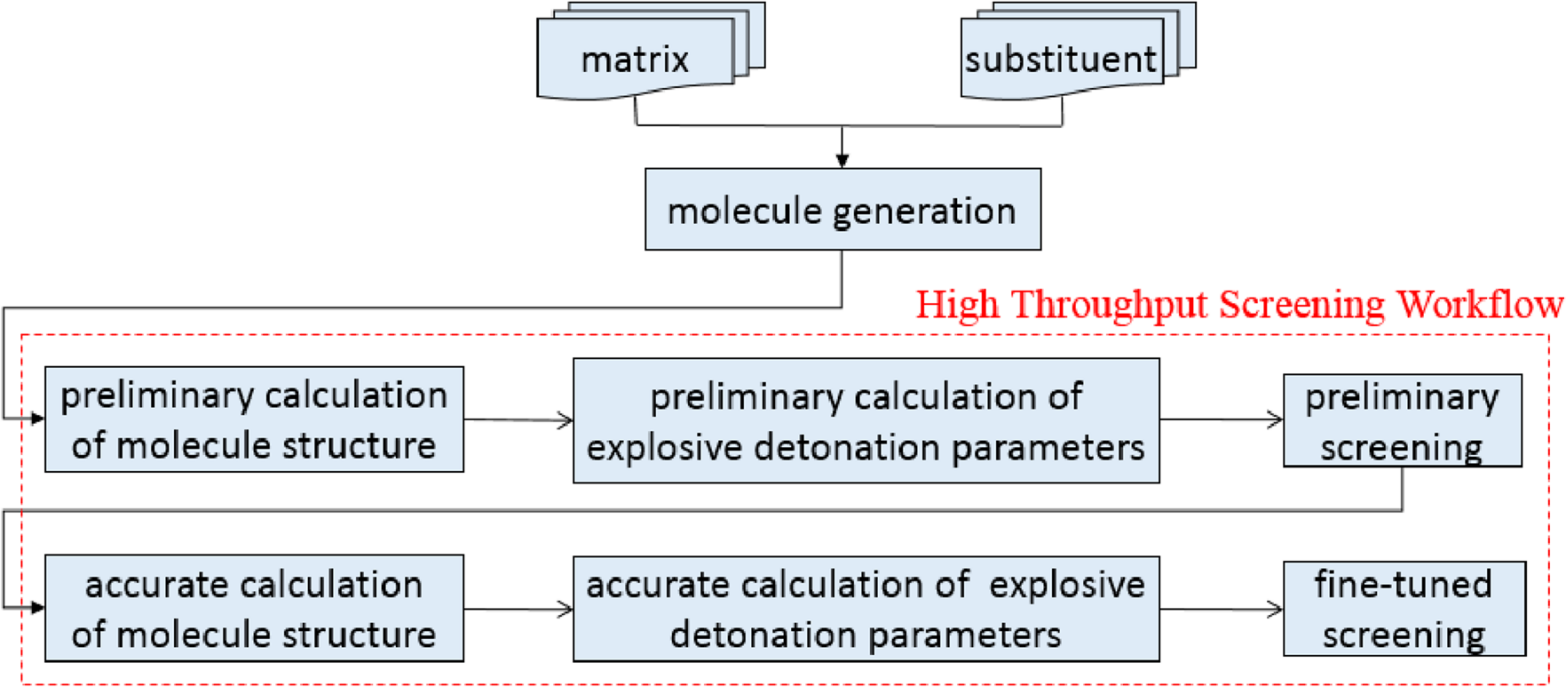
Molecule screening process.

The molecule screening process is illustrated in Fig. 6. Firstly, with the matrix and a variety of feasible substituents specified by energetic material designers as inputs, a large number of possible molecule structures are generated and optimal structures are obtained with energy minimization calculations; Then, for each structure, a series of preliminary but fast calculations are carried out to get the approximate explosive detonation parameters, and a set of molecule structures that meet the explosive detonation performance requirements are selected and saved to the database. After this coarse screening process, the number of the feasible molecule structures is greatly reduced. Then the process of ”calculating molecule structure—calculating explosive detonation parameters” is performed again for the selected molecule structures in the database with more accurate but time-costly calculations and the final molecule structures that meet the requirements are obtained. This two-step screening process makes a balance between the efficiency and accuracy.

### Customization of components

Prior to customization of the workflow for the molecule screening process introduced above, it is necessary to further sort out the sub-processes involved, and build a component for each sub-process based on the underlying software programs or tools as well as the execution information. Following the procedures introduced in Section “Interactive customization of components”, the components used are listed in Table 1.

**Table 1:** Components in the molecule screening process.

Component	Software	Input	Output
GenMatrix	GenMatrixTool	Matrix structure database	Matrix structure file
GenMoleStruct	GenMoleStructTool	Molecule structures for Matrix and substituents	Structures of candidate molecules
CalcMoleStruct	CalcMoleStructTool	Molecule structures and necessary parameters	Log file
CalcDetonParam	CalcDetonParamTool	Log file	Denotation parameters
SaveDB	CouchDB client	Summary of denotation parameters	Records in database
ScreenMole	CouchDB client	Conditions for molecule screening	Satisfactory structures

### Construction of workflow

**Figure 7:**
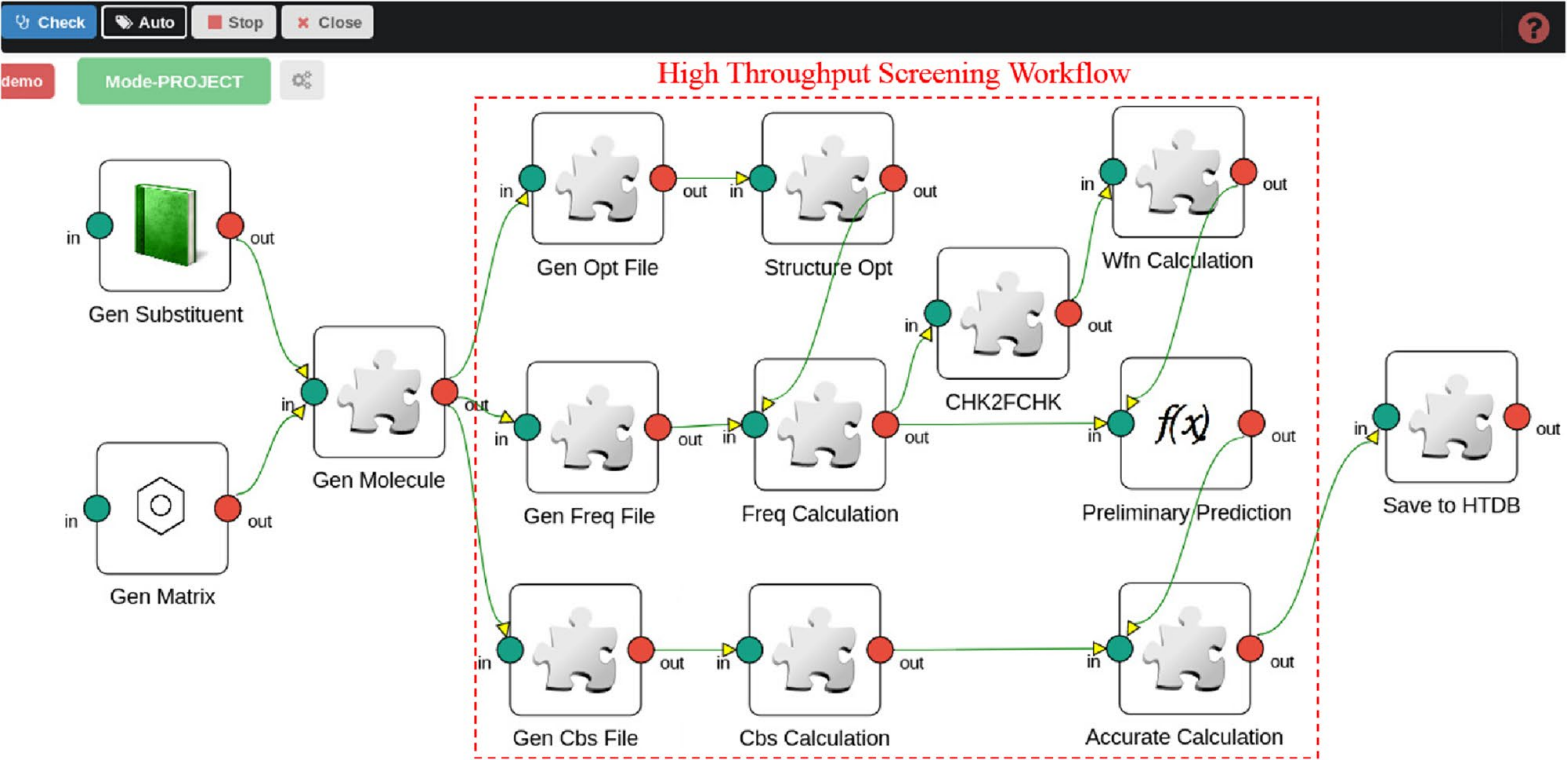
Workflow for screening of energetic molecules.

According to the detailed sub-processes and the customized components for the molecule screening process, the workflow is constructed with HSWAP following the procedures introduced in Section “Interactive customization of workflow”, which is depicted in Fig. 7.

### Construction of integrated platform with GUI

**Figure 8:**
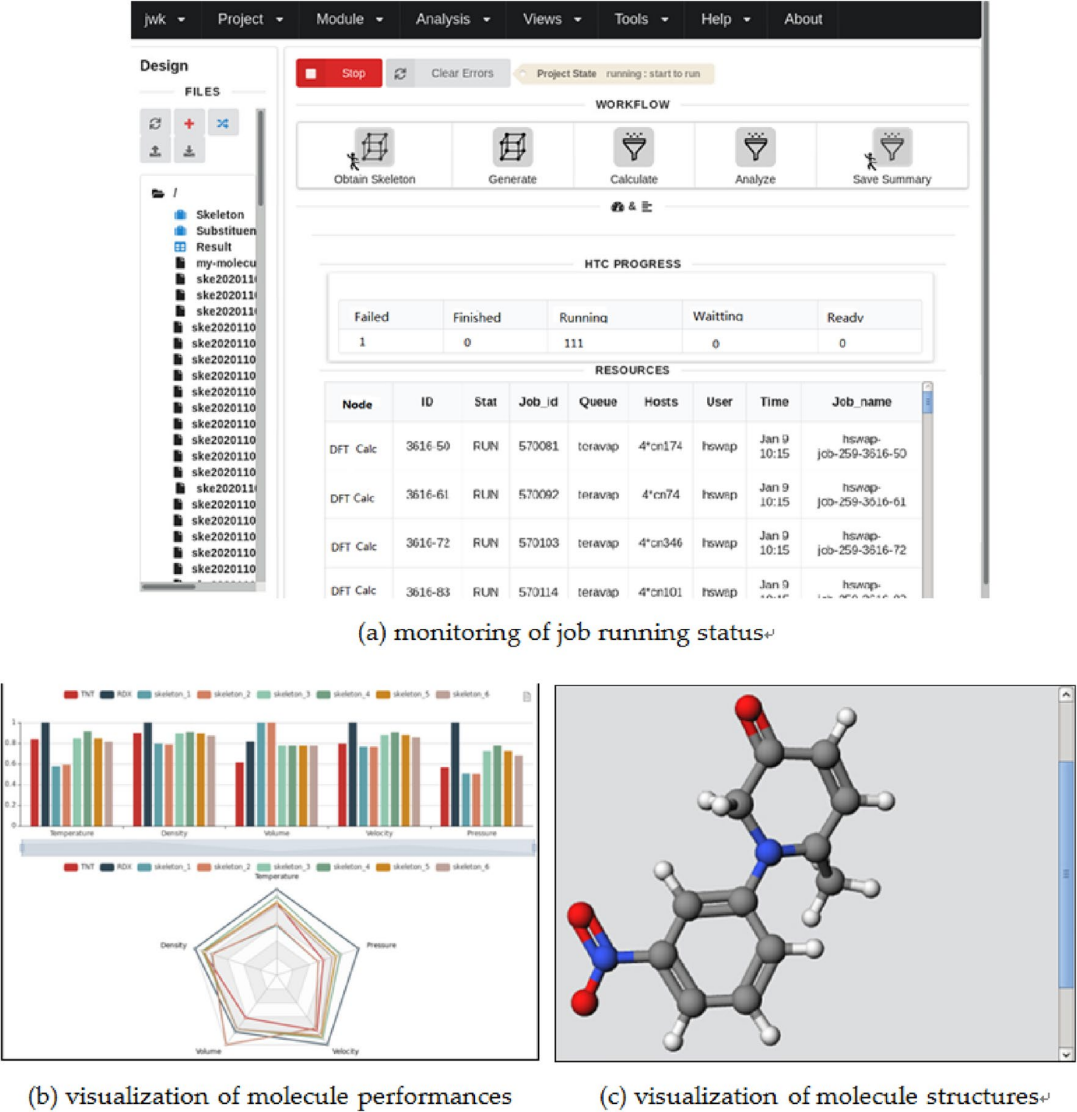
GUI of the integrated platform for molecule screening.

To enable designers to perform the screening process of energetic molecules with ease, an integrated platform with GUI that adapts to usage habits of domain users is designed and implemented, as shown in Fig. 8. The platform contains direct access to the main sub-process, for example the generation of molecule structures could be conducted by the molecule construction module, and the screening of molecule structures with satisfactory explosive detonation performance from a large number of feasible molecules could be conducted by the quantitative calculation module. In addition, the platform also provides the functionalities of visualization of the calculation results and molecule structures, as well as the monitoring of the running status of the workflow, offering a convenient way for energetic material designers to visualize and analyze the screening results.

### Example

**Figure 9:**
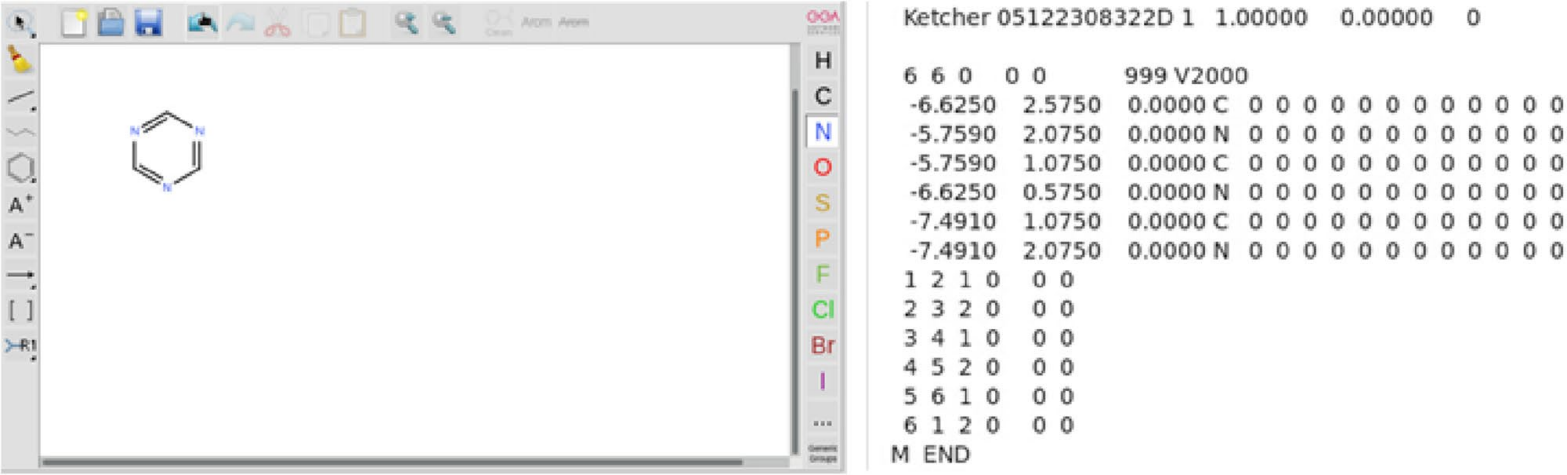
Generation of the molecule structure for the matrix.

In this section a simple example is given for demonstrating the main procedures for the screening of energetic materials using the developed platform. First, build the skeleton of the matrix with the canvas for matrix generation. In this example a typical matrix for energetic molecules, namely 1, 3, 5 triazine, is selected. As illustrated in Fig. 9, the sketch of the matrix could be interactively drawn in the canvas by replacing the three C atoms in the benzene with N atoms. Once completed, a formatted file containing the atomic positions and bond connectivity of the matrix is automatically generated.

**Figure 10:**
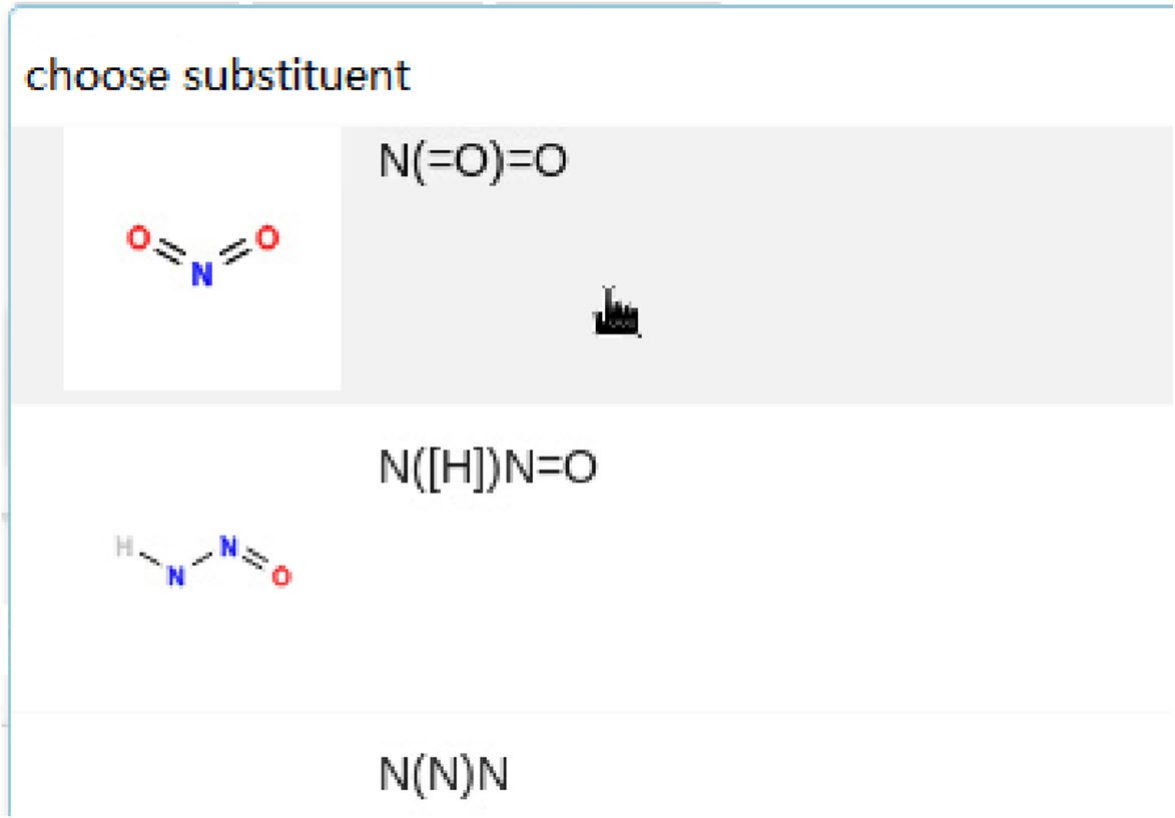
Chosen of the substituents.

After the selection of the matrix, a set of substituents could be chosen from the substituent library. For simplicity only the nitryl is used, as depicted in Fig. 10. The maximum number of substitution positions also needs to be specified, which is set to 2.

**Figure 11:**
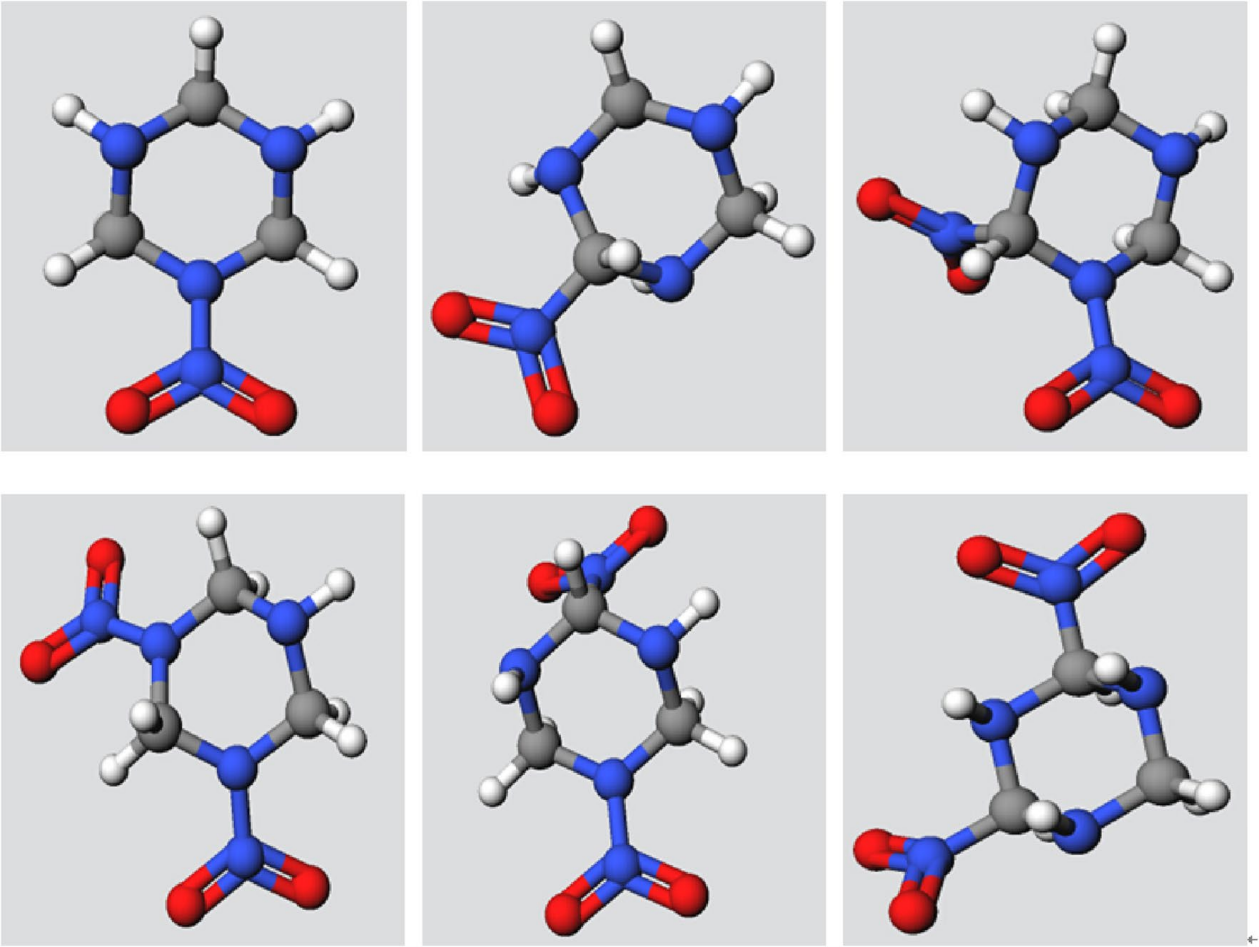
Structures of candidate molecules to be screened.

The molecule structures of the selected matrix and the substituent are then transferred to the successor component as the input for building the candidate molecules to be screened. At the back end of the platform, the encapsulated software behind the component is called to find the feasible combinations of the matrix and the substituents, complete the molecule structures by adding missing hydrogen atoms and remove duplicated molecules, yielding a set of 6 candidate molecules, as shown in Fig. 11. It is worthwhile mentioning that the screening process is naturally a high throughput process in actual engineering case. The throughput, which is the total number of the candidate molecules to be screened, may be as high as tens of hundreds. In this example, for the simplicity and clarity of the demonstration, the matrix and the substituents are chosen such that the throughput of the screening is only 6, which means only 6 candidate molecules are to be screened, nevertheless the screening process introduced in the example is the same as the practical screening process with much larger throughput.

Following the workflow described in Section “Construction of workflow”, these candidate molecules are then transferred to the subsequent component that is responsible for optimization of molecule structures based on the density functional theory. For each candidate molecule, an optimization job is carried out to find the optimized parameters such as the atomic positions, the bond angles and the atomic charges. This batch of jobs are submitted to a high performance computing cluster and run in parallel. Scheduling of the related software and hardware computing resources are handled automatically.

**Figure 12:**
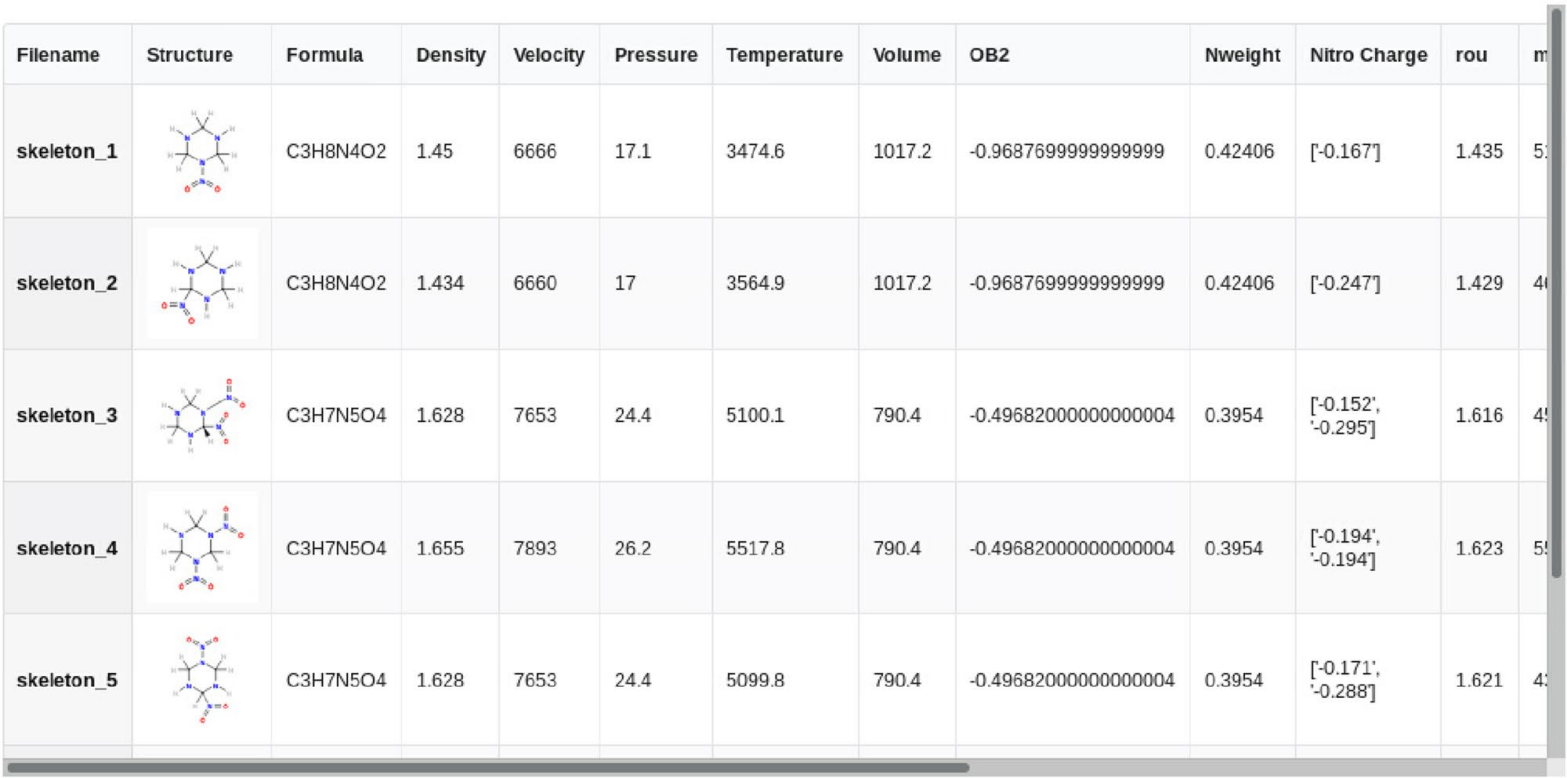
Calculated detonation parameters of the molecule to be screened.

Similarly, a batch of frequency calculations and wave-function analysis are per-formed to evaluate the molecule properties including the vibrational spectra, the orbital occupation, and the charge and energy distribution. With these properties and some empirical equations of state, key detonation parameters of the candidate molecules, such as the detonation temperature, detonation velocity and detonation pressure are preliminarily estimated. In the case with large number of molecules to be screened, a notable portion of candidates with poor detonation performances may be eliminated for efficiency purpose. Detonation performances of the remaining ones are further evaluated with more accurate but costly DFT calculations, which could be finally extracted and comparatively viewed as shown in Fig. 12.

It could be noticed that with the help of this integrated platform, users only need to specify the necessary information of the matrix and the substituents, the execution of simulation tasks and the transmit of data between connected components are automatically handled without user interruption, which greatly improved the efficiency of the whole screening process. Nevertheless, there also exist some limitations for applying the proposed integrated platform for actual engineering work. One major limitation is the unbearable time consumption. Although with high throughput screening techniques many simulation tasks could be conducted in parallel, the total simulation time for a complete screening process could be several days as some simulation tasks such as accurate DFT calculations are very time consuming. To accelerate the simulation process, reduced-ordered surrogate models or data-driven models based on machine learning techniques may be applied to replace the time costly accurate simulations.

The reliability of numerical simulations lies on the accuracy of the simulation results. In this screening process, the reliability of a single simulation task is guaranteed by the software or the tool used in the simulation. For example, the reliability of DFT calculations of the potential energy, the energy band and the charge distribution is guaranteed by the DFT software, which is usually well tested through a validation and verification procedure. In addition, as an integrated platform, extensive tests are made to make sure that the simulation tasks in the workflow are executed orderly as expected and data between components is correctly transmitted. However, the errors or uncertainties of numerical simulations may arise from many aspects. For example, the inaccurate empirical models, the inaccurate model parameters may also introduce uncertainties in the final results, which may be studied in our future work.

## Conclusions

Numerical simulation has long been a standard approach for accelerating the discovery of novel materials with satisfactory performances. To screen the molecular components and structures of materials with best performances from molecule spaces of large capacity, high throughput screening process are conducted, whereby a large amount of feasible molecules are built from given molecular skeletons and functional groups, and corresponding performances are evaluated. For given candidate molecule, to comprehensively predicts the relationship between molecular components, structures and corresponding performances, a series of costly numerical tasks needs to be conducted in parallel on supercomputers, which requires extensive collaboration of various data processing tools, modeling and simulation software programs with close dependence of data and control flow, as well as the automatic scheduling and execution of interconnected tasks in super computing environment. To facilitate an efficient molecule screening process, in this paper a specialized integration simulation platform is constructed based on the scientific computing workflow engine HSWAP. The main procedure for the construction of the platform is introduced in detail. The platform frees domain users from specialized knowledge of scheduling and execution of batch tasks in super-computing environment, and error-prone process of management of the collaborations of a series of computing software and data, leading to huge improvement of the screening efficiency.

## Data Availability

The datasets used or analysed during the current study available from the corresponding author on reasonable request.
